# Metropolitan Hospital Sunday Fund

**Published:** 1904-12-24

**Authors:** 


### METROPOLITAN HOSPITAL SUNDAY FUND.

### ANNUAL GENERAL MEETING.

At the Mansion House on Friday, 16th instant, the annual general meeting of constituents of the Metropolitan Hospital Sunday Fund was held, the Lord Mayor (Mr. Alderman John Pound), president and treasurer, presiding. The attendance of members of the Council and representatives of the congregations was larger than usual.

The formal resolution "That the report of the Council for the year 1904 be hereby received and approved" was moved by Mr. Robert Grey (treasurer of the Foundling Hospital). Sir William Broadbent in seconding said the report (which has already been published in The Hospital) was a record of a year's hard and conscientious work. There was no question which came before the Council which was not carefully considered from every point of view, and the decisions come to were not merely the thoughtless work of officials. He referred particularly to the Distribution Committee, which dealt with the claims of every institution, and the decisions of which all met with the hearty approval of the Council.

### The City Orthopædic Hospital.

Alderman Sir John Bell, while approving generally the work of the Committee, was sorry that he must dissent from its decision in the matter of the City Orthopædic Hospital, from which a grant had been withheld. That institution had been in existence over 50 years, and its only crime was that it refused to amalgamate with another hospital. He had never been in the hospital, but he had read a pamphlet on the question by Earl Maitland, its chairman, and he had come to the conclusion that the Committee had exceeded the bounds of justice in withholding the customary contribution. There was nothing in the laws of the constitution which justified that action. Judging by the photographs in the pamphlet it was not the fact that the hospital was not properly ventilated and suitable for its purpose. It was intended for the use of dwellers in the East End, and it was much more conveniently situated for them than the one at the West End. He believed that the *Lancet* endorsed all he had said, and he thought the Management Committee were quite right in preferring to remain where they were on their own freehold than to be transferred to leasehold premises at the West End. Why the hospital should be depleted of the usual award of £250 he failed to see, and he hoped the matter would be reconsidered and that the institution would be given a chance of carrying on the good work it had done for so many years.

### The Question of Contribution to Medical Schools.

The Hon. Stephen Coleridge then rose to move as an amendment to the resolution—

> That in the opinion of the Council of the Metropolitan Hospital Sunday Fund no moneys of the fund should be assigned to any hospital which contributes to the support of the medical schools or laboratories from its general fund for the relief of patients.

This amendment was, he said, identical with a resolution adopted by the League of Mercy. He regretted the absence through illness of the Archdeacon of Westminster, who had intended to second the amendment, but had sent him the following letter:—

> "May I ask you kindly to express to the meeting my profound disappointment at being prevented by illness from seconding the resolution to be proposed by you? It is of the utmost importance in the interest of the institutions themselves that money given in church for the relief of patients should not be used to support medical schools and laboratories. The assurance given to us last year by the Lord Mayor, upon which assurance we withdrew our amendment, was not, in my opinion, substantiated by the subsequent statement of the Hon. Secretary of the Hospital Sunday Fund. I trust that this year you will insist upon the matter being made so clear that there is no room for misunderstanding.—I am, &c., Basil Wilberforce."

Mr. Coleridge explained that the Archdeacon and he had understood from what they were told at last year's meeting of the Sunday Fund that it had been the custom for the Distribution Committee to deduct from the grants made to each hospital the amount given by it to its medical schools. Investigation showed, however, that this was not in fact the practice of the Committee, and he hoped there would be no such mistake on this occasion. There was, he contended, a very grave responsibility resting on the Council as trustees of a fund subscribed by the beneficent in church for the sole purpose of providing attention for the sick poor, and the diversion even of a penny of that fund for any other purpose was a practice that all honourable people must regard as reprehensible, and one that ought to be stopped. But many of the great London hospitals had taken part of the money given them and had handed it over to institutions which published no accounts, and what became of that money he could not tell. It might go to swell the emoluments of the professors, or it might go to lower the fees of the students. He had made inquiries and found it was not applied to the latter purpose, so it must either go to the professors or to the support of a practice which many of them believed to be displeasing to God. He contended very strongly that that was a diversion that ought not to take place. He was glad to see that Sir Henry Burdett was present that day, because he was happy to think that on this question of the perversion of funds from the purposes for which they were subscribed they were able to see as one man. Sir Henry had said:—

> It is manifestly wrong that any hospital committee should devote to the purpose of medical education any portion of the funds subscribed by the public for the relief of the sick.

And again:—

> I do most heartily hope that the public will combine as one man and set their face absolutely against this perversion of the funds of the hospital to the struggling school attached to it.

He therefore hoped he might count on the very weighty support of Sir Henry Burdett on that occasion. In conclusion he would remind the meeting that he represented in this matter a large number of thoughtful people who were trying to do their duty in life; but they all knew what were the results to people who, however conscientiously, ventured to criticise those who managed great public institutions.

The Rev. Lionel S. Lewis, of All Saints', Buxton Street, which he described as quite close to the London Hospital, seconded the amendment. It fell to his lot, he said, to visit the poor of that neighbourhood, and to see them in their own homes. From Mr. Stephen Coleridge he had learned, with indignation, that since the Hospital Sunday Fund had granted money to the London Hospital, the London Hospital had paid over £32,000 to its medical school. But, as he was made painfully aware, there was not sufficient accommodation in the London Hospital for the sick poor; and when he saw the breadwinners of poor families unable to get the attention they needed, and learned that funds subscribed for their relief were diverted for other purposes, he felt as one felt when one saw a woman struck or a child ill-treated. He believed the plea put forward in extenuation was that since that money had been diverted many more students had come to the London Hospital, and that good had been done in that way. But did anyone expect that £32,000 could be spent and no good at all result? That did not make it morally justifiable. He learned also from Mr. Stephen Coleridge that a sum of £7,000 had been spent by the London Hospital out of its general funds on a sports ground for the students. He thought that showed a lamentable want of sense of proportion in the minds of the people who voted the money for that purpose when it was well known that for £4 or £5 a very good football field could be hired which would answer every purpose. On behalf of the sick poor of London he protested against such action. He considered that those responsible had failed in their trust, and that they should no longer be given grants from that Fund unconditionally.

The Hon. Sydney Holland said that as chairman of the London Hospital he thought he might ask their indulgence and claim also to be just as much interested in the sick poor as others who had spoken. Indeed his whole life was given up to working for the sick poor—he said it in no spirit of boasting, but as a matter of fact and of common knowledge—and he could, therefore, afford to let pass the uncharitable sneers which had been levelled at him. He did not know the London Hospital was to be attacked on the question of its medical college, and he certainly thought that as the Commission appointed by the King's Fund was now dealing with this very matter its consideration by that meeting was premature and inconvenient. He would, however, deal with the specific points that had been raised. As to the £7,000, he considered that since the clergyman who had mentioned it lived so close it would have been more generous had he asked him about it. He would have told him that, having money to invest and considering the land a good investment, they had bought it at a cost of £7,000, and they could sell it to-morrow for £10,000. At present they were getting 4 per cent. interest on their money.

This statement was received with cheers, during which Mr. Holland turned to Mr. Lewis.

"I beg your pardon?" he said interrogatively; and then ironically, "Oh! I beg your pardon; I thought you apologised. I'm sorry for having done you that wrong"—a sally which was received with some amusement.

Continuing, Mr. Holland went on to say that Mr. Coleridge and Mr. Lewis called it wrong to give money to medical colleges because that money was subscribed for the sick poor. But in his opinion the best way to help a hospital to do its work well was to have an efficient college in connection with it. It was true that at the London they contributed £2,500 to the school, but they got over £11,000 worth of work done for nothing in return. The poor in the hospital benefited because they had a good staff. Not being endowed they could only pay their professors the salary of third-rate clerks, but the students paid them fees for teaching, and in that way the poor had the benefit of the services of the most eminent men of the day. As to vivisection, there was practically no vivisection at the London Hospital.

Mr. Coleridge: It is registered as a place for vivisection under the Act.

Mr. Holland: Yes; and that is why you hate it! Continuing, he said there was no progress made at hospitals where they had no school. At the Poplar and Tilbury Hospitals, of which he was also chairman, they were content to do their best for cases as they came in; but at the London every disease of the body was carefully studied with a view to the discovery of its cause, and of means for prevention and cure. They had a bacteriological department, a clinical department, and a laboratory, all kept up by the school, without which the expense would be far greater. He resented the suggestion that those who objected to vivisection were the only honourable men, and asserted that the hospitals need not be alarmed at Mr. Stephen Coleridge's diatribes. The grants of money were authorised by the people who subscribed the money, and it was only those who did not subscribe who objected. He had asked such people to subscribe to hospitals without schools, but they still refused, and in his opinion this was merely an excuse. Let them remember that part of every sovereign spent on hospitals went for secretaries' salaries, for clerks' wages, for scrubbing and cleaning, and what not. People subscribed to carry on the whole work of the undertaking, and because they came and saw how it was being done. Mr. Coleridge and his friends, however, seemed to make it their object to do all the harm they could to the London Hospital.

Mr. Coleridge objected to that statement. He wanted to reform the London Hospital. He had sent them a subscription, and it had been returned.

Mr. Holland said Mr. Coleridge's sovereign had been returned with thanks, because they knew it could not have been sent to do good to the hospital. Mr. Coleridge had stated that the medical college published no accounts; but he was wresting the meaning of the word "publish." Accounts were most carefully kept; they were printed and audited, and any subscriber could get a copy.

Dr. Glover appealed to the meeting not to allow itself to be misled by the arguments of Mr. Coleridge, which went to the root of the progress of medicine in the Victorian age—progress that had made that age an epoch. He instanced the case of the Middlesex Hospital, which for 150 years had opened its doors to cancer, yet in all that time what had been done? Now in our laboratories they were trying to get at the root of the disease and so find a cure. He also referred to the benefits derived from the use of diphtheria serum, and declared that Mr. Coleridge had no right to try to tie the hands of medical men fighting against disease.

Dr. Reinhardt, an old student of the London, said he was sure the students would be prepared to pay for facilities for using clinical material. One large hospital—the North London or University College Hospital—received from its school at the present time a considerable amount in fees. Medical men would always be found ready to give their services in return for the benefits they enjoyed. It was quite well known that their incomes and their positions were the result of the facilities the hospitals afforded them.

### Why the Question had Arisen.

Sir Henry Burdett said he would not have risen had his name not been mentioned, but he should like to make his position clear. He differed from Mr. Sydney Holland in thinking that the medical schools had a claim to any part of the funds subscribed to the hospitals for the relief of the sick poor. As a parent with four sons, he felt that he could not put a boy of his into the medical profession with satisfaction if he knew that any portion of the money he ought to pay as a parent for his education was practically diverted from moneys given for the sick poor. He knew of no other profession where such a question could arise; and until 1897 no such question as this with regard to the medical schools could have arisen in London. Why, then, had it arisen? Not because the hospitals had been placed in funds through the King's Hospital and Sunday Funds, but because the number of entries at the London medical schools had seriously fallen off during the last seven years, and as a consequence they had become poorer. As the number of students became less the competition became greater, and greater efforts were necessary to maintain the medical schools. There was no doubt that

London was behind the times in not possessing one great medical school. The sooner the present system was ended, or mended, the better, for it was a system of small competing schools. The professors were paid inadequate sums, and so they merely had gentlemen who worked hard in practice and devoted a part of their time to medical education.

A Remedy for the Future.

It was his opinion that if the medical schools required more money, and if it was felt and thought that hospitals to which schools were attached should be supported by the public, then the remedy was a simple one. All the hospital authorities which favoured this view had to do was to open a special fund and ask the public to contribute on a statement which set forth quite clearly the purpose for which the money was required. (Hear, hear.)

Hospital Finance.

It was quite impossible to hope that the public would go on increasing their subscriptions to voluntary hospitals indefinitely unless those hospitals faced the question of expenditure. He found from researches he had been conducting during the year that the present rate of expenditure exceeded the present rate of contribution, though so noble had been the response of the public to the hospital appeals that the sum given in the last ten years had increased by more than the cost of the requirements of the increased population. In other words the increase of the hospital population had been greater than the increase of the whole population of London. For that reason and on the grounds he had stated he hoped the consideration of the amendment would be adjourned for another year. The Commission appointed by the King's Fund, consisting of Sir Edward Fry, Lord Welby, and the Bishop of Stepney, was engaged in going into the whole question of the relation of the hospitals to their medical schools. Before the next meeting of constituents they would have the report of that Commission, and then would be the time to deal with the matter. (Hear, hear.)

Mr. Coleridge said that in response to the appeal addressed to him by Sir Henry Burdett he asked leave to withdraw his amendment. He would like it to be on the understanding that he hoped next year he would be met in a rather different spirit from that displayed by his old friend Mr. Sydney Holland.

The report was then adopted.

The Council was formally re-elected, with the addition of the Right Rev. Bishop Amigo, of Southwark; the Rev. C. J. Proctor, Vicar of Islington; Sir James Thomson Ritchie; and Mr. Deputy Wallace, to fill vacancies. A vote of thanks to Mr. George Herring for his continued generosity was adopted; June 25th was fixed for Hospital Sunday, 1905; an obsolete law, No. X., referring to the initial establishment of the Hospital Sunday Fund, was deleted; and the proceedings terminated with a vote of thanks to the Lord Mayor for presiding.

